# Editorial: Psychological factors in physical activity for healthy life and healthy aging

**DOI:** 10.3389/fpsyg.2023.1128555

**Published:** 2023-03-22

**Authors:** Raquel Vaquero-Cristóbal, Lucía Abenza-Cano, Estélio Henrique Martin Dantas, Pablo Jorge Marcos-Pardo

**Affiliations:** ^1^Facultad de Deporte, UCAM Universidad Católica de Murcia, Murcia, Spain; ^2^Active Aging, Exercise and Health/HEALTHY-AGE Network, Consejo Superior de Deportes (CSD), Ministry of Culture and Sport of Spain, Madrid, Spain; ^3^Nursing and Biosciences Post-graduation Program, Doctorate of Federal University of State of Rio de Janeiro, Rio de Janeiro, Brazil; ^4^Biosciences Laboratory of Human Movement (LABIMH), Tiradentes University, Aracaju, Brazil; ^5^Neuropsychological Evaluation and Rehabilitation (CERNEP) Research Centre, Scientific Projects Organization and Research Training (SPORT) Research Group (CTS-1024), Department of Education, Faculty of Education Sciences, University of Almería, Almería, Spain

**Keywords:** aging, health, interventions, physical activity, psychology

The increase in the number of older people worldwide, as well as the increase in life expectancy that has occurred in recent years, has led the World Health Organization (WHO) to recently proclaim that we are in the decade of Healthy Aging. The programs promoted under the umbrella of this program not only focus on the older people, but on all population groups, in order to reach this stage in the best health conditions, understanding the concept of health as encompassing the physical, psychological and social aspects. To this is added the need to contemplate the psychological and motivational aspects in physical activity programs for health.

As our populations age around the world, it is imperative to ensure that older people are able to age as healthy and active participants as possible. Chronic diseases are major health problems for older people as they age into the foreseeable future. The identification and implementation of effective non-pharmacological preventive strategies is an important public health priority, and many of these strategies will need to be implemented in the primary care setting. Currently, known effective interventions are multicomponent and multidimensional intervention programs including strength, aerobic, flexibility and balance training, cognitive exercises, nutritional education, motivation and other domain-specific interventions. There is a need to better understand the optimal approaches to multi-domain interventions.

The focus of this Research Topic was to increase knowledge on the interaction between psychology, physical activity and health, and more specifically we focused on the benefits of physical activity practice on psychological aspects and health in populations at different stages in the pursuit of healthy aging; on the use of motivational strategies and self-determination theory to improve adherence to healthy physical activity programs; and on the relationship between psychological factors and other physical or social variables related to health.

This collection of studies provides a series of evidence on the efficacy of physical exercise interventions on the physical, psychological, social and emotional health of exercisers and can offer some guidance to health professionals in primary care centers and collegiate physical activity educators. It is necessary to continue working to educate from prevention at all ages in order to be able to age with health and have an active and healthy aging. This also means educating all health professionals working in the community and in primary care settings to understand their role, and working together with collegiate physical activity educators to prevent, reduce and even reverse many chronic diseases that benefit from multi-domain interventions.

Specifically, this collection of articles contribute to increase the limited body of literature focused on the consideration of psychological factors as a fundamental part to consider in healthy physical activity programs. Strength training, multi-component training, mental health, visual attention, cognitive training, intrinsic motivation, life satisfaction and quality of life are topics covered in this collection of articles. Thus, the authors come from different countries, reflecting the global interest in this topic.

In this way, this Research Topic covers topics as varied as the effects of combined strength and cognitive training or strength exergaming to improve cognitive or functional outcomes in adults and older adults (Esmaeilzadeh, Kumpulainen et al.), the effects of physical exercise or multicomponent exercise programs on the mental health (wellbeing, anxiety and depression) and cognitive functions of older adults with/without dementia who live in a nursing home and do/do not require wheelchair assistance (Da Silva et al.), the effects of physical exercise on the quality of life of healthy older adults (Wei et al.), the effect of an attributional retraining (AR) intervention designed to increase control-related outcomes in a physical activity context for older adults with compromised health (Parker et al.), the effect of physical exercise on the life satisfaction among college students (Zhang et al.), motivational predictors of physical activity and sedentary behaviors in adults and older-adults (Esmaeilzadeh, Rodriquez-Negro et al.), the preferences of older-adults in terms of marketing healthy habits (Wang et al.), or the gender differences in fatigue levels among professional drivers using the BAlert app, smartphone app that approaches exhaustion with psychophysiological measures (De La Vega et al.).

We hope you enjoy reading the articles included in this Research Topic and that you find them useful for your professional development in the pursuit of healthy aging.

## Author contributions

All authors listed have made a substantial, direct, and intellectual contribution to the work and approved it for publication.

